# Facile Synthesis, Static, and Dynamic Magnetic Characteristics of Varying Size Double-Surfactant-Coated Mesoscopic Magnetic Nanoparticles Dispersed Stable Aqueous Magnetic Fluids

**DOI:** 10.3390/nano11113009

**Published:** 2021-11-09

**Authors:** Saurabh Pathak, Rajni Verma, Prashant Kumar, Arjun Singh, Sakshi Singhal, Pragati Sharma, Komal Jain, Rajendra Prasad Pant, Xu Wang

**Affiliations:** 1 Department of Mechanical Engineering, University of Melbourne, Parkville, VIC 3052, Australia; 2 School of Engineering, RMIT University, Melbourne, VIC 3001, Australia; swatiinsp@gmail.com (P.S.); xu.wang@rmit.edu.au (X.W.); 3 School of Physics, The University of Melbourne, Parkville, VIC 3010, Australia; 4 School of Sciences, RMIT University, Melbourne, VIC 3001, Australia; prashantkhichi92@gmail.com; 5 Academy of Scientific and Innovative Research, CSIR-NPL Campus, New Delhi 110012, India; arjunsmy6@gmail.com (A.S.); komaljain90@gmail.com (K.J.); head.irmcsirnpl@gmail.com (R.P.P.); 6 Institute of Nuclear Medicine & Allied Sciences, DRDO, Brig SK Mazumdar Road, Delhi 110054, India; sakshisinghal534@gmail.com

**Keywords:** ferromagnetic resonance, magnetic nanoparticles, Fe_3_O_4_, spin dynamics, Langevin fitting

## Abstract

The present work reports the synthesis of a stable aqueous magnetic fluid (AMF) by dispersing double-surfactant-coated Fe_3_O_4_ magnetic nanoparticles (MNPs) in water using a facile ambient scalable wet chemical route. MNPs do not disperse well in water, resulting in low stability. This was improved by dispersing double-surfactant (oleic acid and sodium oleate)-coated MNPs in water, where cross-linking between the surfactants improves the stability of the AMFs. The stability was probed by rheological measurements and all the AMF samples showed a good long-term stability and stability against a gradient magnetic field. Further, the microwave spin resonance behavior of AMFs was studied in detail by corroborating the experimental results obtained from the ferromagnetic resonance (FMR) technique to theoretical predictions by appropriate fittings. A broad spectrum was perceived for AMFs which indicates strong ferromagnetic characteristics. The resonance field shifted to higher magnetic field values with the decrease in particle size as larger-size MNPs magnetize and demagnetize more easily since their magnetic spins can align in the field direction more definitely. The FMR spectra was fitted to obtain various spin resonance parameters. The asymmetric shapes of the FMR spectra were observed with a decrease in particle sizes, which indicates an increase in relaxation time. The relaxation time increased with a decrease in particle sizes (sample A to D) from 37.2779 ps to 42.8301 ps. Further, a detailed investigation of the structural, morphological, and dc magnetic properties of the AMF samples was performed. Room temperature dc magnetic measurements confirmed the superparamagnetic (SPM) characteristics of the AMF and the *M*-*H* plot for each sample was fitted with a Langevin function to obtain the domain magnetization, permeability, and hydrodynamic diameter of the MNPs. The saturation magnetization and coercivity of the AMF samples increased with the increase in dispersed MNPs’ size of the samples. The improvement in the stability and magnetic characteristics makes AMFs suitable candidates for various biomedical applications such as drug delivery, magnetic fluid hyperthermia, and biomedicines.

## 1. Introduction

Aqueous magnetic fluids (AMFs) have emerged with great potential due to their substantial development in the field of biomedical applications [1,2,3]. The preparation of magnetic fluids (MFs) has engrossed the interest of researchers due to the tremendous potential of the development of active controlled devices at low power [4,5,6,7,8]. MFs have sensation and actuation properties that drive their interdisciplinary applications through hybrid materials [8,9,10]. MFs are stable colloidal suspensions of mesoscopic magnetic particles (MNPs) (diameter ≈ 2–30 nm) dispersed in the desired carrier liquids. MFs are usually constituted by three components: MNPs, surfactants, and liquid carrier medium [11,12]. The choice of MNPs is made to meet the requirement of the desired magnetic moment and viscosity. Soft magnetic materials are the ideal candidates for MFs’ preparation as they provide magnetization and demagnetization characteristics in the presence and absence of externally applied magnetic fields [13,14]. The size and morphology of the MNPs significantly affect the magnetic moment of the materials. The decrease in the size of the MNPs results in a decrease in the magnetic moment; however, it improves the stability [15,16]. Further, the increase in size results in a higher viscosity, which induces more frictional losses, reducing the overall performance of the systems. Thus, a trade-off between the stability and magnetic moment is key for the selection of the size of the particles [11,17]. The surfactant is used to prevent agglomeration in the system, and chosen based on the size of the MNPs to provide the desired steric repulsion [18]. Oleic acid is the most commonly used surfactant; it provides a chain length of around 2 nm, which is ideal for the MNPs of average size 10 nm [19]. The choice of carrier liquids is entirely based on the application requirement. A wide variety of carrier liquids, such as water, kerosene, silicon oil, ethylene glycol, hexane, etc., have been used and their selection is based mainly on the temperature range, viscosity, biocompatibility, and interaction with MNPs [20]. The selection of the ideal constituents’ composition is vital to meet the requirements of the particular applications and achieve the desired performance. 

MFs display unique properties such as ultra-low friction, self-levitation/sealing, magnetic bearing formation, sticking to the magnet, enhanced thermal conductivity, variable density, and viscoelasticity [21,22]. The motion of the MFs can easily be controlled by an external magnetic field. In the absence of an external magnetic field, MF displays a Newtonian behavior. Whereas it shows a two-fold shift with the application of field and displays non-Newtonian characteristics [23,24]. The viscosity of these fluids remains constant at fixed temperature and does not depend on external factors such as applied shear strain, loading, oscillation, or angular frequency in the absence of the applied field [25]. However, with the application of an external magnetic field, the viscosity of MFs changes significantly and they show a shear-thinning behavior in general, with the application of a static or dynamic loading [11]. At a low applied load, MFs behave like semi-solids as MNPs form chain-like structures in the field direction and these structures restrict the smooth streamline of the fluid [9]. With the increase in external loading, chain structures in MFs start to break, resulting in a decrease in viscosity and they behave like a liquid [18]. This decrease in viscosity of MFs with an applied external loading is termed shear-thinning behavior. From the device point of view, the magneto-viscosity of the materials is tuned in such a way that it utilizes the applied loading (both rotational and oscillation) range that is producing the free stream-thinning viscosity in the MF [26].

The stability of the AMFs can be improved by providing an additional layer of surfactants on the MNPs’ surface to stipulate an apparent double-layer stabilization. The second surfactant layer is attached just opposite to the primary surfactant of the MNPs. Their non-polar head is connected to the MNPs and the polar head attaches with aqueous media [20]. The type of surfactant and its quantity significantly affect the structure and stability of the AMFs as demonstrated by Petrenko et al. through small-angle neutron scattering (SANS) studies [27]. AMFs have generated substantial interest due to their biomedical applications such as biosensing, antibacterial activities, magnetic fluid hyperthermia treatment, magnetic resonance imaging (MRI) targeted drug delivery, cell separation, and bioimaging application [28,29,30]. AMFs present many advantages such as their low toxicity, excellent temperature, chemical stability, biocompatibility, and superparamagnetic (SPM) nature, which makes them significantly desired material [31,32,33]. However, agglomeration, oxidation, and uniformity are the major issues of AMFs that require immediate attention [34,35]. The major effort in the current biomedical applications is focused on improving the stability and dispersivity in biocompatible liquids such as water and optimizing the constituents to improve the magnetic characteristics that provide better control [36,37,38]. 

Moreover, from the literature, we can conclude that wet chemical synthesis is the better processing method for AMFs. It generally results in low impurity concentration and highly stable fluids can be prepared with incredibly low processing difficulties [39]. The prime focus of our research is on preparing stable AMF with uniformly distributed MNPs to achieve isotropic properties of the fluid. Cao et al. [40] synthesized AMFs using the chemical coprecipitation method by coating three different surfactants (sodium dodecyl sulphate, oleic acid, and polyethylene glycol) to investigate the effect of different surfactants on the stability of the AMFs [40]. They confirmed from the transmission electron microscopy (TEM) results that the MNPs coated with sodium dodecyl sulphate and oleic acid showed a low stability due to agglomeration. Furthermore, the polyethylene-glycol-coated MNPs showed a higher stability due to a lower agglomeration. However, the long-term stability and the effect of gradient magnetic field have not been probed, which are the main factors that reduce the stability of AMFs [40]. Dai et al. [41] prepared the MNPs by a low-temperature freeze-drying technique followed by a coating of 3-aminopropyltriethoxysilane (APTES) on the surface to form amino-functionalized MNPs [41]. To prepare a stable water-based fluid, they attached graphene oxide (GO) to amino-functionalized MNPs by electrostatic adsorption to obtain GO-functionalized MFs (GO-NMPs) composite [41]. Petrenco et al. [27] reported the preparation of AMFs stabilized by sodium oleate (SO) and dodecylbenzene sulphonic acid (DBSA). They correlated the properties of the AMFs with varying surfactant types both in the presence and absence of externally applied magnetic field. Both DBSA and SO produced a micelle structure and interacted with the surfactant concentration, which resulted in an aggregation of MNPs and a large and fractal-type aggregation observed in the prepared AMFs [27]. Morales et al. [42] synthesized MNPs coated with oleic acid and pluronic-polymer-based aqueous dispersion. They suggested that the oleic acid has a low hydrophilic–lipophilic balance, which yields a low stabilization. Thus, they used a block copolymer to enhance stability. However, the prepared aqueous fluid had a lower stability due to the long-term instability of the polymer [42]. Further, Bica et al. [43] probed the effect of various surfactants combination of different chain lengths (lauric acid (LA), myristic acid (MA), oleic acid and DBSA) on the stability of the AMFs. They reported that the double layer combination of both LA and MA resulted in a higher stability and in improvement of the biomedical application [43]. Even though there are numerous research efforts dedicated towards the preparation of AMFs with high stability and magnetization, a single AMF cannot be treated as best performing fluid for all applications. Various applications demand different aspects (viscosity, density, and other physicochemical properties) of the AMF in different ranges, which makes probing the effect of individual constituents of AMFs vital for high-performance fluids [44,45]. 

The investigation of the spin dynamics of the magnetic materials is very important considering it provides necessary information about the physical mechanisms in magnetic materials [46]. FMR is an exceptional characterization tool for probing the microwave spin resonance characteristics of MNPs systems. FMR provides key information about the MNPs system, such as its relaxation behavior, spin glass transition, dipolar interaction, exchange interactions, anisotropy effects, dead layer, core-shell structure, and magnetic domains of the systems [47]. In addition, other important phenomena such as the magnetic state of the system (ferromagnetic (FM) or SPM), low-temperature carrier freezing, and spin canting can also be studied using FMR. One of the major advantages of these systems is that the particles in both a dry as well as in a fluid state can be characterized by changing the sample stage [15]. FMR is a very versatile and sensitive instrument, and it can sense the FMR signals in the range 10^14^–10^16^ magnetic moments/minute in ordered FM materials [19]. It has a sensitivity of the relaxation times of the magnetically polarizable particles in the range 10^−7^–10^−10^ s. Further, it can also perceive the paramagnetic impurities in the material as minor as the ppm level [48].

Magnetic manipulations and control over the properties give exciting opportunities for the development of active control and high-performance devices. In this work, we focus on the development of a stable, homogeneous, biocompatible, and high magnetic strength fluid. The AMF is prepared by dispersing Fe_3_O_4_ MNPs in water. The stability of the dispersion is achieved by using two layers of surfactants in reverse directions to each other to allow cross-linking. The stability of the prepared AMF is probed by rheological measurements both in the absence and presence of the externally applied magnetic field. Generally, the stability and magnetic properties in AMFs are conflicting properties as the increase in size results in an increase in magnetic strengths of the AMFs; however, their stability decreases. Thus, the optimization of the size of dispersed MNPs is vital. The static and dynamic magnetic characteristics of the AMF are investigated by a vibrating sample magnetometer (VSM) and FMR measurements. The experimental results are correlated with various theoretical predictions to calculate various parameters such as spin concentration, domain magnetizations, permeability, spin–spin relaxations, and hydrodynamic diameter. Further, the structural investigations of the prepared Fe_3_O_4_ MNPs are carried out by X-ray diffraction (XRD) measurement. Moreover, a Rietveld refinement of the patterns is done to calculate various structural parameters. We prepare four AMF samples with varying size distributions. The morphology and size distribution of all the AMF samples is performed by TEM and small-angle X-ray scattering (SAXS) measurement. The prepared AMF shows enhanced stability, which will be vital to improving the performance of existing applications, as well as lead to the development of other novel applications. The performance of the fluids remains the key bottleneck in AMF-based applications. The present work focuses on solving the grand challenge associated with biomedical applications of AMFs, i.e., technical issues related to the synthesis of AMFs for homogeneous, biocompatible, high magnetic strengths and stable MFs in large-scale production.

## 2. Synthesis and Characterization of AMF Samples

**Figure 1:**
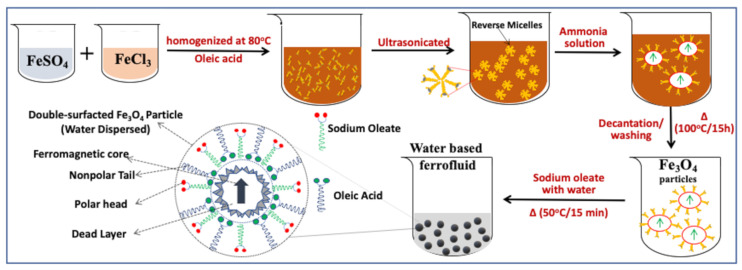
Schematic representation of the aqueous magnetic fluid preparation by dispersing double surfactant (oleic acid (blue string) and sodium oleate (green string)) Fe_3_O_4_ magnetic nanoparticles using two-step wet chemical synthesis method (adapted from [20] with permission from Elsevier, 2019.

The synthesis of a stable AMF is a state-of-art technique as it requires the optimization of several counteracting forces. We have implemented a wet chemical synthesis approach for the preparation of AMF consisting of two steps. First, oleic-acid-coated Fe_3_O_4_ MNPs were prepared by the surface-modified chemical coprecipitation method. In this method, reverse micelle structures of oleic acid allow the growth of particles inside, preventing agglomeration [16,17,18,21,39]. In the second step, the oleic-acid-coated Fe_3_O_4_ MNPs were dispersed in water using the secondary surfactant sodium oleate. The schematic representation of the typical synthesis process adopted is depicted in Figure 1 [20]. For the synthesis of Fe_3_O_4_ MNPs, salt precursor solutions of Fe^3+^ and Fe^2+^ were prepared in the stoichiometric ratio of 2:1 and mixed under a constant stirring rate for 30 min. Afterward, 8 mL of oleic acid was added to the solution which formed reverse micelle structures upon constant stirring and heating at 80 °C, followed by ultrasonication for 15 min. A 25% concentration ammonia solution was then added to the solution for precipitation [4,9,28,49]. Four different samples were prepared by adding different amounts of ammonia solution to maintain a fixed pH. The respective samples corresponding to pH 8, 9, 10, and 12 were named as A, B, C, and D. The resultant solution after ammonia addition was maintained at 80 °C under constant stirring (600 rpm) for 60 min to allow the growth of the particles. The resultant solution was filtered using a NdFeB permanent magnet and washed several times using water and ethanol. The washed particles were dried in a vacuum oven overnight to remove any trace of water. A homogeneous solution of sodium oleate was prepared by adding 3.8 mg of sodium oleate to 20 mL of water and heating it at 50 °C for 30 min under constant stirring. Finally, the oleic-acid-coated MNPs were dispersed in homogeneous aqueous sodium oleate solution by manual stirring [20]. 

The detailed characterization of the AMF comprises the determination of stability, crystalline purity, structural, morphology, rheological behavior, and static/dynamic-magnetic properties by various sophisticated analytical characterization tools. The stability of the AMF was calculated by a parallel plate magneto-rheometer (MCR-301 with MRD 70 setup, Anton Paar, GmbH, Ostfildern, Germany) with a transverse magnetic field (1.2 T). The gap optimization and standard calibration were performed using silicon oil standard with PP-20 spindle. For structural and phase identification, an XRD (Ultima-IV, Rigaku, Tokyo, Japan) technique was used followed by a Rietveld refinement analysis for a detailed crystallographic modeling. Further, the crystallite size of the prepared MNPs was obtained by the Debye–Scherrer (D-S) formula and Williamson–Hall (W-H) method. For obtaining the morphology and size distribution of the samples, SAXS (Rigaku Ultima-IV with rotational stage and liquid geometry) and TEM (model TECNAI F30, FEI, Hillsboro, OR, USA) were utilized. The size range of the Fe_3_O_4_ MNPs was calculated by the SAXS by fitting the experimental data using Nanosolver software (3.7.6.0, Rigaku, Tokyo, Japan) assuming a Gaussian or log-normal distribution. The static magnetic properties were calculated by VSM (model 7410 VSM, Lakeshore, OH, USA). Further, the spin dynamics of the AMFs were probed using an EPR spectrometer (EMX-10, Bruker, Berlin, Germany) using a 100 kHz field modulation. The AMF samples were investigated using an X-band (9.85 GHz) microwave frequency, and a TM011 mode cavity. The FMR spectra of all the AMF samples were recorded at room temperature and different microwave spin resonance parameters such as resonance field, peak-to-peak line width (ΔH), g-value, spin–spin relaxation time, and spin concentration were calculated by fitting the FMR spectra. To avoid any distortion to the FMR spectra of the sample, the amplitude of the modulation was kept lower than one-third of the ΔH. The FMR profile shape was further correlated to the effect of varying size distribution on the super-exchange and dipolar interactions of the MNPs.

## 3. Results and Analysis

### 3.1. Stability Analysis of AMFs Using Magneto-Rheometer

**Figure 2:**
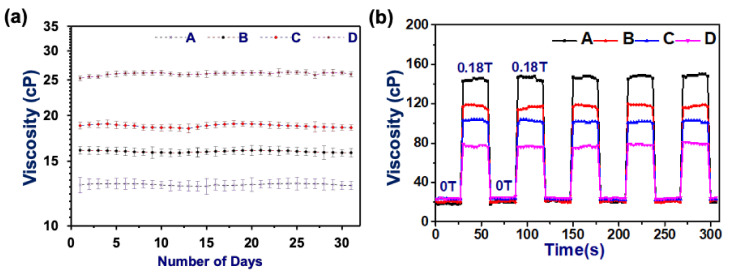
(**a**) Viscosity of AMF samples in the absence of magnetic field for 30 days measured by calculating the viscosity each day (average of ten measurements). The flat curve demonstrates the stability against gravity. (**b**) Viscosity vs. time response of all the samples with magnetic field ON (0.18T)/OFF(0T) consecutively for five cycles. The viscosity response to the applied gradient field shows that the viscosity almost regains the same values, which highlights the stability of the AMFs.

AMFs are required to remain suspended and do not agglomerate with the application and removal of the magnetic field. The stability of the AMFs was probed both in the absence and presence of an applied magnetic field (gradient field) ON (0.18T)/OFF (0T) at constant temperature (25 °C). In the absence of a magnetic field, the AMF shows a Newtonian behavior and the viscosity remains constant [11]. The viscosity of each AMF sample was obtained at the fixed temperature in constant shear mode (100 s^−1^) by taking the average of 10 measurements for each day (till 30 days). Figure 2a shows the viscosity plot of all the AMF samples, which demonstrates that the viscosity remains almost constant over the period of 30 days. This shows that the prepared AMF samples are highly stable for a prolonged period and do not settle down due to gravity. The sampling process is maintained similarly for all the samples and the system is calibrated using standard silicon oil [17,18]. The viscosity of the sample with lower size distribution (AMF sample (D)) is higher, whereas with an increase in size distribution, the viscosity decreases in the absence of a magnetic field. This happens due to greater interparticle friction between the particles with the reduction in size [21,39,50,51]. 

In the presence of a magnetic field, the viscosity response completely reverses, and larger-size particles AMFs (sample A) show a higher viscosity. This can be related to their higher magnetic moment which responds more strongly to the applied magnetic field and has stronger dipolar interactions between the MNPs [52,53,54,55]. The stability of the AMFs in the gradient magnetic field was obtained by probing the AMF samples with a continuous magnetic field ON/OFF. The viscosity versus time response has been obtained for all the samples with magnetic field ON (0.18T)/OFF (0T) with a 30 s interval. From Figure 2b, it is evident that the viscosity remains the same even after multiple cycles of gradient field. This confirms that the MNPs are not agglomerating under the gradient magnetic field and show almost reversible characteristics when the magnetic field is removed. In the presence of the field, MNPs align in the direction of the magnetic field, forming a chain-like structure which results in an increase of the viscosity of the AMF [11,21]. The stability analysis using a gradient magnetic field confirms that the AMFs are showing completely reversible behavior between Newtonian to non-Newtonian [53,56]. Moreover, the long-term stability has been confirmed for the 30-day viscosity calculation, in which the AMFs are showing a Newtonian behavior and a constant viscosity at a fixed temperature. 

### 3.2. Structural Analysis of Aqueous Magnetic Fluids Using X-ray Diffraction Method

**Figure 3:**
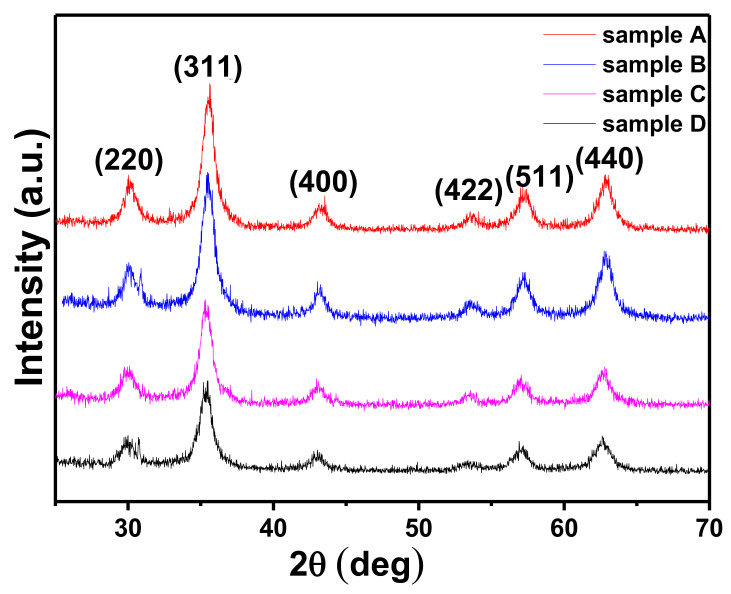
XRD pattern of the AMF samples (A, B, C, and D) confirming single-phase spinel ferrite structure with no additional peaks.

**Figure 4:**
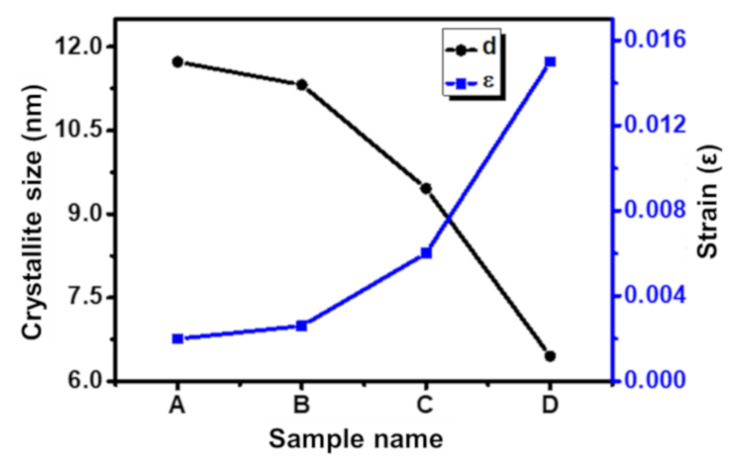
Variation of crystallite size and induced strain for different AMF samples prepared at different precipitation pH.

The structural investigation of the MNPs is vital as it provides information about the qualitative phase, preferred growth orientation, structural parameters, crystallite size, strain, lattice parameter, and percentage crystallinity of the material. The structural and phase identification of all the AMF samples was carried out by the XRD technique. The diffraction patterns of all the samples were recorded in 2θ range 20°–80° with a step size of 0.01° using a Cu-K_α_ source (λ = 1.5406 Å) at slow scan (0.5°/min) [4,49]. The XRD plot of all the AMF samples prepared from the coprecipitation technique (A, B, C, and D) is shown in Figure 3. The peak positions in all the XRD plots perfectly match with the single cubic spinel ferrites structure. The diffraction peaks observed for sample A at 30.54°, 35.83°, 43.38°, 53.86°, 57.37°, and 63.10° perfectly match with the lattice planes of spinel ferrites, (220), (311), (222), (422), (511), and (440) (JCPDS card no-22-1086), respectively [15,17,39]. The other samples (B, C, and D) also have peaks at the same position. Some minor shifts are present which can be correlated to the anisotropy, sampling, and instrumental factors [8,9,28,48]. From Figure 3, we can conclude that all the AMF samples are single cubic spinel phase and their full width at half-maxima (FWHM) increase from sample A to D consistently. This systematic increase in the FWHM corresponds to the decrease in the crystallite size from samples A to D [39,46,57]. The crystallite size and the strain in the AMF samples were obtained by the D-S formula (Equation (1)) and W-H method (Equation (2)) [4,48]:

(1) $$d=\frac{k\lambda }{\beta_{hkl\;}cos\theta }$$

(2) $$\beta_{hkl}cos\theta =4\epsilon sin\theta +\frac{k\lambda }{d}$$

where, *d*—crystallite size, *k*—Scherrer constant, *β_hkl_*—FWHM corresponding to each peak, *θ*—Braggs’ angle, *ε*—crystalline strain. The obtained values of the crystallite size and induced strain from Equations (1) and (2) are summarized in Table 1 [21,46,48]. The crystallite size obtained from both the D-S and W-H methods is in close proximity. The crystallite size of the samples decreases with an increase in precipitating pH of the samples. The crystallite size decreases from 10.6 nm to 5.8 nm for samples A to D systematically. This confirms that the crystallite size of the material synthesized using the coprecipitation method decreased with an increase in reaction pH while other synthesis parameters were kept constant [20,49]. The increase in pH results in a significant increase in nucleation sites, which restricts the long-range growth in the material resulting in smaller crystallites [4,9,58]. The variation in D-S and W-H methods appears due to the instrumental and strain broadening. The induced strain for samples A and B is 0.0020 and 0.0026, respectively, which is lower compared to samples C and D which have higher induced strain values of 0.006 and 0.015, respectively. Moreover, it is evident that the decrease in size of the materials generally results in higher strain and more impurities [4,59,60]. Figure 4 shows the variation of crystallite size and strain for all the samples. The lattice constant (a) was also calculated for all the samples by taking all the diffraction planes; it lies in the range 8.3482 to 8.3440 Å, which is in good agreement with the standards (8.342 Å) [16]. The decrease in lattice constant from samples A to D is due to the large ionic radii difference of Fe^2+^ (0.77 Å) as compared to Fe^3+^ (0.67 Å) [11,15]. With the decrease in size, the cation distribution changes, and Fe^3+^ and Fe^2+^ ions are replaced with each other from their respective tetrahedral (A) and octahedral (B) sites. This results in a deviation of the lattice constant from the standard values. The cation distribution at A and B sites plays a vital role in governing the structures of these AMFs [48,49].

**Figure 5:**
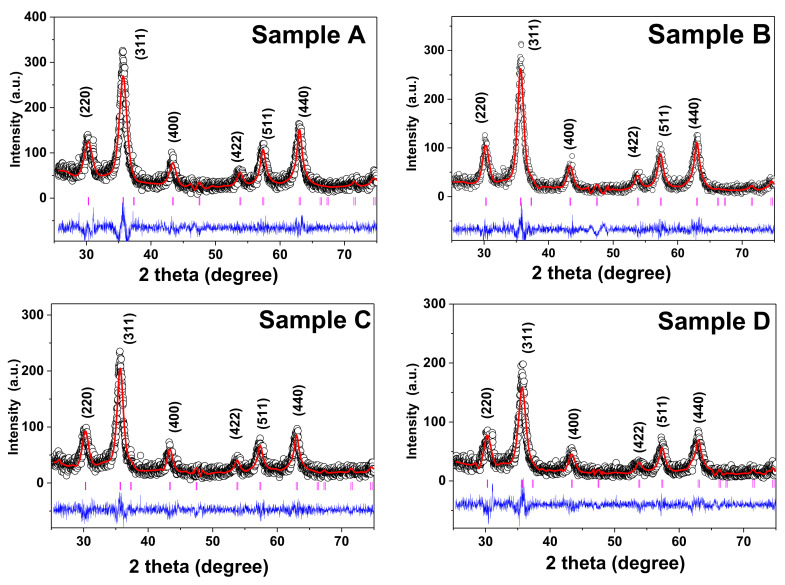
Rietveld refined XRD patterns of the AMF samples (**A**–**D**). The open circles show the experimental data and the solid lines show the refined XRD patterns. The blue line at the bottom shows the difference between the observed and calculated patterns and the vertical standing lines shows different Braggs’ position.

Afterward, XRD patterns obtained for all the AMF samples were analyzed using the Rietveld refinement technique using Fullprof Suite software. Various structural factors such as atomic position, site occupancy, and crystallographic factors have been obtained from the refinement. All the XRD plots were refined using the *Fd*-3m(227) space group of spinel structure [48]. The Rietveld refined patterns of all the AMF samples are shown in Figure 5 and different structural parameters are summarized in Table 1. The observed data from the experiments are shown in open circles, the calculated pattern is shown as the solid line in Figure 5. In addition, the difference between the observed and experimental data is shown at the bottom as a blue line, and the vertical lines show the calculated Braggs’ positions of each plane. This perfect match with the standards shows the crystalline single-phase spinel ferrite group of *Fd*-3m(227) symmetry [46]. The refinement of the XRD pattern of the AMF sample was carried out in two steps. Firstly, the removal of the background and scaling was performed. In the second step, the structural parameters and site occupancy was determined [15]. The refinement of all the samples was carried out by fixing the octahedral sites (16d (1/8, 1/8, 1/8)) and tetrahedral sites (8a (1/2, 1/2, 1/2)) with free oxygen (32e (x, x, x)) sites [50]. All the structural and refinement parameters such as R-factor ((R_F_ = crystallographic factor, R_P_ = profile factor, R_B_ = Bragg Factor, R_wp_ = weight profile factor, and R_exp_ = expected value) are summarized in Table 1. The large reliability factor is observed for the smaller crystallite samples as it leads to the large, diffused scattering compared to that of the highly crystalline sample which dominates the Braggs’ scattering [4]. The refined profile has a goodness of fit (χ^2^) in the range of 1.23 to 1.43, which constitutes a good fit between observed and experimental data. The value of the lattice parameter (a = b = c) and direct cell volume (V) of all the AMF samples were calculated by the refined pattern and are listed in Table 1. 

### 3.3. Size Distribution of Aqueous Magnetic Fluids Using Transmission Electron Microscopy

**Figure 6:**
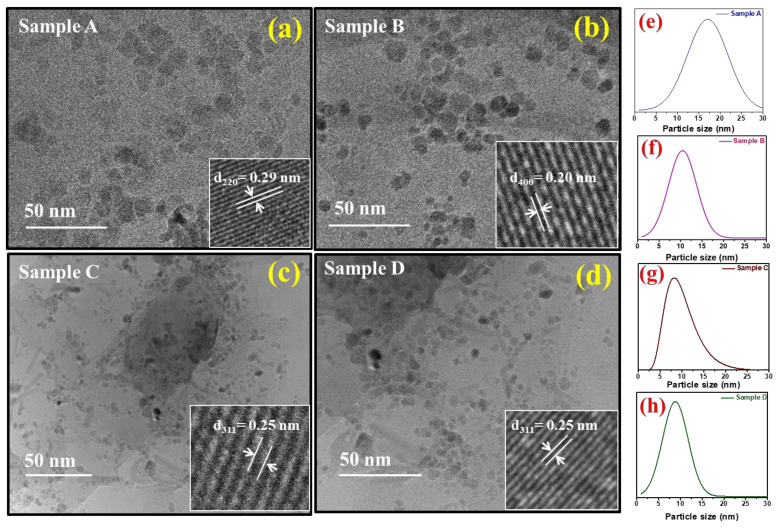
(**a**–**d**) TEM micrographs of samples A, B, C, and D, respectively. Inset (**a**–**d**) HRTEM images showing interplanar distances 0.29 nm, 0.20 nm, and 0.25nm corresponding to planes (220), (400), and (311), respectively. (**e**–**h**) Size distribution graph of samples A, B, D (Gaussian distribution) and sample C (log-normal distribution).

The electron micrographs of the double-surfactant-coated Fe_3_O_4_-based AMFs samples (A, B, C, and D) are shown in Figure 6a–d. The inset figures show the HRTEM images of the respective samples. The samples for the TEM analysis were prepared in the liquid state by diluting it with kerosene, followed by heating from the light source. In all the samples, the particles are almost spherical with equisized distribution. The inset Figure 6a shows the HRTEM image of sample A having an interplanar spacing of 0.29 nm, which corresponds to the (220) plane of the spinel ferrite phase [19]. Similarly, the inset in Figure 6b–d shows the HRTEM image of samples B, C, and D, respectively. An interplanar spacing of 0.20 nm and 0.25 nm is measured in samples B, and C and D, respectively, corresponding to the planes (400) and (311) [4,8,61]. The size distribution of the samples is shown in Figure 6e–h where (e–h) are best fitted with a Gaussian distribution and (g) is fitted with a log-normal distribution [33,62,63,64,65]. The size distribution for sample A is 3–30 nm with an average particle size of 17.2 nm. Similarly, for sample B, the size distribution is 2–22 nm with an average size of 10.2 nm; for sample C, the size distribution is 2–22 nm and the average size is 8.3 nm; and for sample D, the size distribution is 1–17 nm and the average size is 7.5 nm. From the electron micrographs, we observe that all the particles are of very narrow size distribution, which is a key requirement for the AMF samples. In all the samples, the particles are approximately spherical. Moreover, from the TEM analysis, it is evident that the size of the sample is decreasing from A to D with an increase in precipitation pH. This is well in alignment with the XRD results. The reduction in size with the increase in precipitating pH is due to a large number of nucleation sites at higher pH [66].

### 3.4. Size Distribution Using Small-Angle X-ray Scattering Measurement

**Figure 7:**
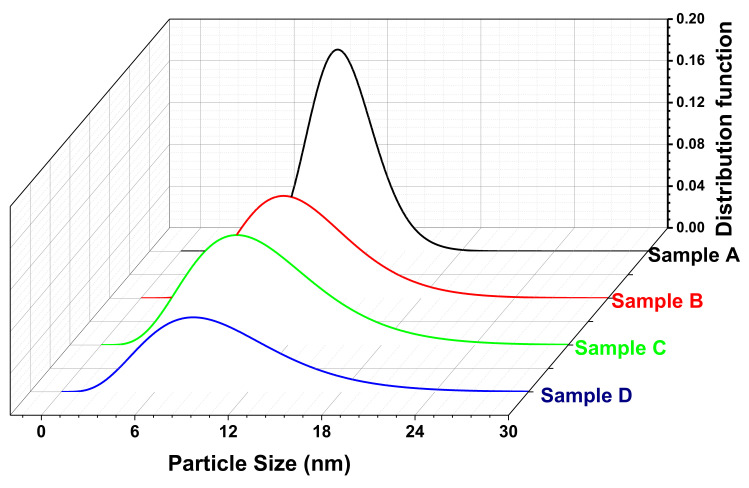
Size distribution plot for the AMF samples (A, B, C, and D) modeled from the SAXS measurements assuming Gaussian or log-normal distribution.

A standard rotational attachment for the liquid capillary sample stage was used for the SAXS measurement of all the AMF samples. The sample was diluted and placed in the very thin capillary (0.07 mm thickness quartz capillary) and sealed from the top. The capillary was provided a slow rotation (330 rpm) for the measurement ranging from 0.05 to 3° with a step width of 0.02° using CuKα radiation. The experimental data obtained from the measurement were modeled to calculate the size distribution of the particles [67,68]. Figure 7 shows the size distribution plot of the samples obtained from Nanosolver software assuming the Gaussian or log-normal distribution. The average particle size obtained from the SAXS measurement with associated resize distribution is shown in Table 2. In addition, the respective values of the size obtained from the TEM are shown for comparison in Table 2 [69,70]. We note that the size of the sample is decreased from A to D and the size distribution for sample A is very narrow but slightly wider for the other samples. The average size of the samples is in good agreement with the TEM results. Although a slight variation in the size distribution from TEM analysis can be well understood, as in the SAXS measurement, a relatively large amount of the sample is under test compared to the TEM measurement. The SAXS results are highly reproducible and reliable [71,72]. The SAXS data modeling using Nanosolver requires various input data such as the density of the materials, interparticle distance, and matrix in which the system is dissolved. SAXS is an immensely powerful and reliable tool for understanding the shape of the sample as well [64,73]. Samples A, B, and D are best fitted with a Gaussian distribution. This indicates that these samples are almost spherical. However, sample C has a left-skewed log-normal distribution which reflects that particles are slightly elongated and differ from the spherical shape [69,70,72]. The SAXS provides a more reliable approximation of the size distribution; however, TEM is a better-suited method for probing the morphology. 

### 3.5. DC Magnetic Measurement of the Double-Surfactant-Coated Fe_3_O_4_-Based Aqueous Magnetic Fluid Sample Using Vibrating Sample Magnetometer

**Figure 8:**
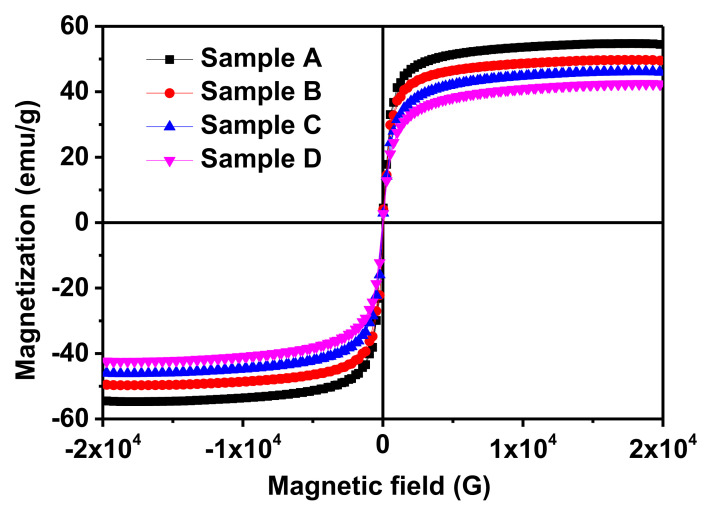
The room temperature *M*-*H* loop of the AMF samples with magnetic field in the range ±2 T dc. The negligible coercivity in all the samples confirms the superparamagnetic nature of the samples.

**Figure 9:**
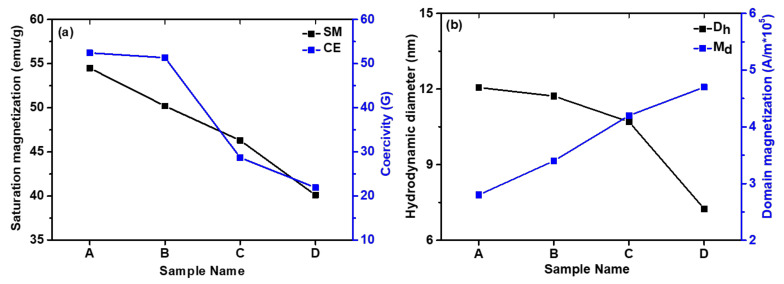
Variation of: (**a**) saturation magnetization and coercivity; (**b**) hydrodynamic diameter and domain magnetization for different AMF samples.

The room temperature magnetic measurement of the AMF samples was performed in the liquid mode using a leak-proof homemade Perspex container with a magnetic field in the range ±2 T dc. This sample holder isolates the sample from the atmosphere so that MF will not interact with the surroundings. The samples were scanned with a magnetic field in the range ±2 T, which is the usual magnetization field range of soft magnetic materials. The *M*-*H* loop of the AMF samples is shown in Figure 8, which depicts the SPM behavior of the AMFs since the coercivity (“CE”-*H_c_*) of all the samples is negligible. The saturation magnetization (“SM”-*M_s_*) of the samples increases with the increase in size, which can be perceived from Table 3 and Figure 8. The SM of sample A is 54.5 emu/g which reduces consistently to 40.1 emu/g for sample D as shown in Figure 9a. The decrease in SM of the AMF samples with a decrease in size results from the presence of a large number of spins occupying the surface of the MNPs [46,67]. The influence of the dead layer becomes more prominent as the size reduces, which also plays a part in the reduction of SM [74]. Table 3 shows the values of the various key magnetic characteristic parameters such as SM, CE, and remnant magnetization (“RM”-*M_r_*), of the AMF samples. Further, the anisotropy constant (*k*_anis_) of the samples was calculated by the formula: *k*_anis_ = $\frac{Hc\times Ms}{0.98}$ [46,48,50,57]. 

The *k*_anis_ of sample A is higher and decreases systematically with the decrease in size of the dispersed MNPs. From Table 3, we observe that the CE and RM of the samples decrease with a decrease in size which is well in alignment with the theoretical predictions and literature. The CE of samples A is 52.44 G which decreases to 21.92 for the smaller size sample D as shown in Figure 9b. Similarly, the RM decreases from 2.18 to 1.27 emu/g for samples A to D. However, the values of the RM and CE are exceedingly small, which clearly indicates that the particles are SPM in nature [4,46,75]. 

**Figure 10:**
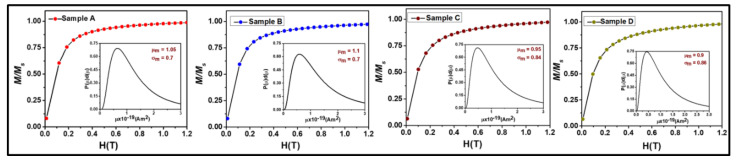
Langevin function fitting of the AMF samples (**A**–**D**) in which symbols represent the experimental data and the solid line represents the fitted curve. The insets of the figure show the permeability and standard deviation of each of the AMF samples.

Further, the *M*-*H* loop of the samples was fitted using the Langevin function given as [4,15,16,46,75]: $M=\int_{0}^{-\infty }L\left(\alpha \right)f\left(D\right)dD-\chi_{i}H$, where *L*(*α*) is the Langevin function{$L\left(\alpha \right)=M_{s}^{f}\left(\coth\left(\alpha \right)-\frac{1}{\alpha }\right)$} with $\alpha =\frac{M_{d}H((\frac{1}{6})\pi D^{3}}{kT}$, $M_{S}^{f}=\emptyset M_{d}$ and *f*(*D*) is the log-normal size distribution of the MNPs that can be expressed as $f\left(D\right)=\frac{1}{\sqrt{2\pi }\sigma_{D}D}e^{\left\{-\frac{\ln{\left(\frac{D}{D_{o}}\right)}^{2}}{2\sigma_{D}^{2}}\right\}}$, where *σ_D_* is the standard deviation, *H* is the applied magnetic field, *k* is the Boltzmann constant, *T* is the temperature, $M_{S}^{f}$ is the magnetization of the fluid, ∅ is the solid volume fraction of MNPs in AMF, $M_{d}$ is the domain magnetization, *D* is the MNPs diameter, *D_o_* is the median diameter and $\chi_{i}H$ is the diamagnetic contribution from surfactant [16]. Assuming a uniform MNPs distribution, the average interparticle distance (*r*) can be given as *r* = (1/*n*′)^1/3^ where $\emptyset =n^{\prime }\left(\frac{1}{6}\pi D^{3}\right)$. The best-fitted raw curves of the samples with the experimental data are depicted in Figure 10, where symbols represent the experimental raw curve and solid line represents the fitted curve [46]. Various deduced parameters and fitting parameters obtained from the Langevin fitting are listed in Table 3. The hydrodynamic diameter (*D_h_*) of the samples calculated from the Langevin function fitting is well in accordance with the particle size calculated by TEM and SAXS and the crystallite size obtained by XRD. The domain magnetization (*M_d_*) of AMFs increases whereas the relative permeability decreases with a decrease in dispersed MNPs. Moreover, we can conclude from the *M*-*H* loop that all the AMF samples are SPM in nature and the SM, CE, RM and anisotropy constant increase with an increase in size of the MNPs. Further, the Langevin fitting of the raw curves has confirmed that the domain magnetization decreases with an increase in size of the MNPs in the AMF samples. 

### 3.6. Room Temperature Spin Dynamics Investigation of Fe_3_O_4_-MNPs-Dispersed AMF

**Figure 11:**
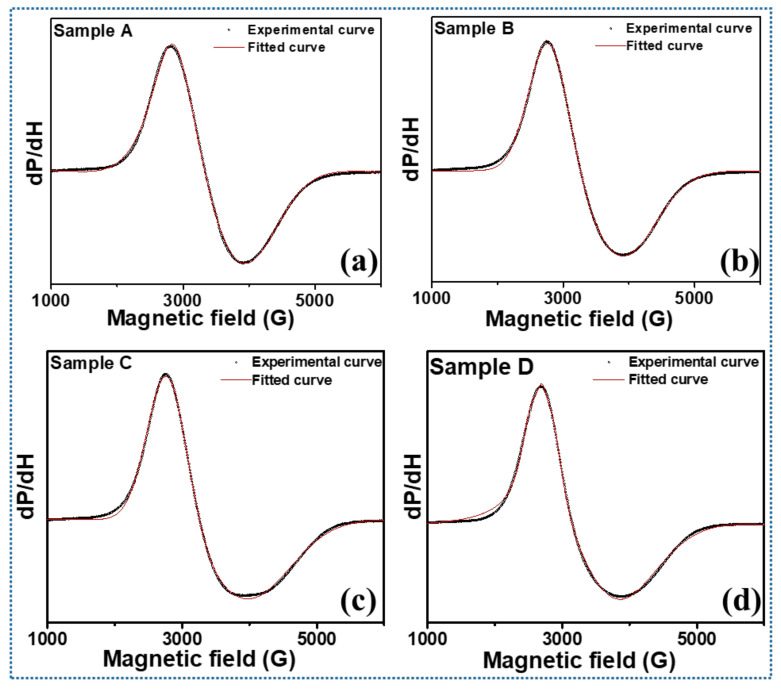
(**a**–**d**) FMR spectra of the respective AMF samples A, B, C, and D with the experimental curve are shown with hollow circles and the solid red lines show the best fit.

The room temperature FMR spectra of the AMFs samples were recorded in a liquid state and the dependence of microwave spin resonance properties on the varying particle size was investigated. The FMR spectra of the samples were fitted with suitable distribution functions and resonance parameters were calculated from the best-fitted curves. The broad FMR spectra of the different AMF samples (Figure 11) with varying MNP sizes confirm the ferromagnetic characteristics of the materials. The experimental data and best-fitted FMR spectra are shown in Figure 11. The FMR spectra clearly show that all the samples are asymmetrical with a lower tail of the broad spectra as compared to the upper tail. This implies that the spins are taking a longer time to relax back to their ground state [47]. Moreover, the broadness of the lower tail increases with a decrease in particle size, which can be related to the higher surface spins inducing anisotropy in the material system [57]. 

**Figure 12:**
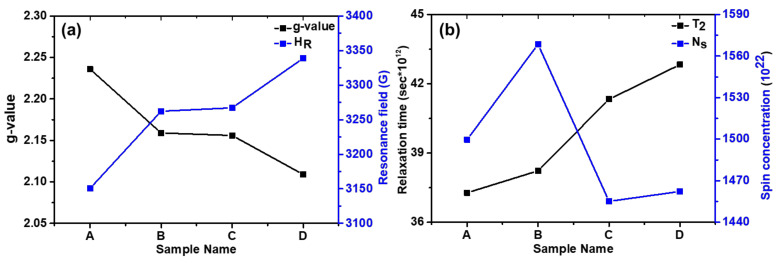
Variation of: (**a**) g-value and resonance field; (**b**) relaxation time and spin concentration of the AMF samples.

The important spin dynamics parameters such as resonance field (*H_R_*), peak-to-peak width (Δ*H_PP_*), Landé g-tensor, spin concentration (*N_s_*), and spin–spin relaxation time (*T_S_*) were obtained for all the AMF samples and are depicted in Table 4. The single broad spectra for all the samples as evident from Figure 11 demonstrate the ferromagnetic behavior of the sample, and no isolated Fe^3+^ and Fe^2+^ exist. The single FMR line spectra are the contribution from the sample and suggest that both Fe^3+^ and Fe^2+^ are in a single phase. Further, the FMR spectra of all the samples are asymmetrical but their H_R_ and Δ*H_PP_* show systematic variations. From Table 4, we observe that the resonance field shifts to higher magnetic field values with the decrease in particle size (Figure 12a). It can be well understood as the larger particle size samples magnetize and demagnetize at lower field due to strong dipolar interactions. This allows the magnetic spins to align in the field direction more easily [19]. The shifting of the resonance peak to a higher value with a decrease in the size of the MNPs can be explained by the magneto-crystalline anisotropy and thermal energy. At smaller crystallite sizes, smaller volumes lead to small magneto-crystalline anisotropy energies, which are expressed as: $E_{A}=K\cdot V$; where *K* is the anisotropy constant and *V* is the volume of the SPM particles [19,46,76,77]. As the anisotropy energy becomes smaller than the thermal energy $E_{T}=K_{B}T$, the magnetic spins start to move freely and randomly in all directions. This leads to an increase in the required magnetic field values to achieve resonance [78,79]. 

Further, the *g*-value is a key parameter of the MNPs sample which provides the orbital contribution to the magnetic moment and is expressed as $g=\frac{h\nu }{\mu_{\beta }H_{R}}$, where *h* is Planck’s constant, *ν* is the microwave frequency, and *μ_B_* is Bohr’s magnetron [80]. The *g*-value increased monotonically from 2.109 to 2.236 with the increase in particle size of the Fe_3_O_4_ MNPs as shown in Figure 12a [46,57]. The *g*-value is an inverse factor of the resonance field and shows just the opposite trend for the magnetic system. Furthermore, a slight incremental change is observed in peak-to-peak linewidth with an increase in crystallite size. The variation in the linewidth is negligible with respect to the magnitude of linewidth itself [19]. Due to regional variations in the local magnetic field experienced by the magnetic spins, a gradation in a magnetic field is experienced by the spins in the ferromagnetic system [48,49,50]. Due to this gradient, these spins achieve resonance at different values of the magnetic field giving a broadness to the FMR signal. As seen in Table 4, the peak-to-peak linewidth increases almost linearly with particle size.

The shape of the FMR signal was analyzed thoroughly through different fitting functions. A Gaussian profile provided the best fit for smaller particle size; however, a transition from Gaussian to pseudo-Voigt was observed in the profile, as the particle size was increased to 10 nm. Pseudo-Voigt is an approximation of a Voigt profile using a linear combination of Gaussian and Lorentzian profiles instead of their convolution [19,39,46,50]. The Gaussian shape of the profiles is indicative of the dipolar broadening in the linewidth. Due to exchange interactions, the dipolar broadening in the center is reduced, leading to a narrowing in the center of the peak and resulting in a pseudo-Voigt shape [19]. Further, the spin–spin relaxation time (*T_s_*) was calculated from the spectral half-width (Δ*H*_1/2_) obtained by fitting the peak shapes and *g*-value, listed in Table 4. *T_s_* is typically expressed using the below Equation (3) [81]:

(3) Ts=ℏgμBΔH12

As the size of MNPs increases, the spin–spin relaxation time shows a sharp decline. A similar decay in transverse relaxation rate has also been reported by Noginova et al. [82]. The spin–spin relaxation time varies with the inverse second power ($T_{S}\propto r^{-2}$, where *r* is the particle radius) of the SPM particles size as suggested by Yin et al. [83]. The relaxation time for sample A is 37.2779 × 10^12^ s; it increases to 42.8301 × 10^12^ s for sample D as shown in Table 4 and Figure 12b. This is also evident from the FMR spectra shape and the lower broad tail. With the decrease in particle size, the thermal energy of the particles becomes dominant, which imparts randomization in the system, contributing to the larger relaxation time [19,39,50]. Further, the spin concentration was calculated using Equation (4) [48]:

(4) $$N_{S}=\frac{9}{4\pi^{2}}\frac{\Delta H_{1/2}}{g\mu_{B}}$$

where Δ*H*_1/2_ is the full width at half-maximum of the absorption peak. It is seen from Table 4 that initially, the spin concentration increases with a decrease in the particle size. However, after a critical point, it starts decreasing (Figure 12b). This unusual behavior of the spin concentration can be understood from Equation (4) as *N_s_* depends on the two key parameters, *g*-value, and Δ*H*_1/2_. Both these quantities have a different behavior with the particle size variation. Thus, the spin concentration of the AMF samples is maximized at the critical point and the maximum spin concentration of 1568.5 × 10^22^ is observed for sample B. This is evidence that the optimization of the size of the MNPs dispersed in AMF plays a key role in governing their performance.

## 4. Conclusions

A double-surfactant-coated AMF was synthesized and a detailed investigation of its stability, structure, dc magnetic properties, and spin dynamics was carried out. AMFs have showed a tremendous potential recently due to their diversified applications in the scientific and technological fields. However, the stability of AMFs remains the key bottleneck in applications as MNPs do not disperse well with water. Herein, we have successfully prepared a highly stable AMF by coating two layers of surfactant on Fe_3_O_4_ MNPs using oleic acid and sodium oleate. The polar head of the oleic acid attaches to the MNPs whereas the polar head of the sodium oleate attaches to water and their nonpolar heads cross-linked to each other allowing a stable dispersion of MNPs in water. The stability of the AMF samples was investigated by a novel approach using a magneto-rheometer and probing the fluid samples in rotational mode at gradient magnetic fields. All the AMF samples showed a high stability against a gradient magnetic field for a prolonged period.

The structural investigation of the AMF samples was carried out by an XRD analysis and the obtained XRD patterns of each sample were probed by the Rietveld refinement method. The crystallite size of the samples varied from 10.6 to 5.8 nm for samples A to D obtained by the W-H method. The structural refinement confirmed the spinel ferrite phase (*Fd*-3m geometry, space group 227). A good fit between the observed and experimental data was perceived with χ^2^ values in the range 1.23 to 1.43. Further, the morphology of the Fe_3_O_4_ MNPs was probed by a TEM analysis, which confirmed the spherical shape of all the samples. The average size obtained for the samples decreased with an increase in precipitation pH consistently (17.2 to 7.5 nm for samples A to D, respectively). Moreover, the size distribution was further confirmed by the SAXS measurement, and the results of the average particle size obtained for sample A was 16.6, which decreased to 7.2 nm for sample D. Further, the SAXS curve gave an indication about the shape of the particles. Samples A, B, and D showed a spherical nature, whereas sample C showed a slightly elongated particle shape. The values of the average size obtained for all the samples using TEM and SAXS were in close proximity and also matched with the trend observed for the crystallite size. 

Further, the dc magnetic measurement of all the samples confirmed the SPM nature and various parameters such as SM, CE, RM, and *k_anis_* were calculated by an *M*-*H* plot. The SM of the AMF samples systematically increased with an increase in size. Further, the raw curve of the *M*-*H* was fitted with the Langevin function, and various parameters such as domain magnetization, permittivity, and hydrodynamic diameter were obtained. The spin dynamics of the AMF samples was carried out by an FMR measurement which confirmed the ferromagnetic nature of all the samples. The resonance field increased with decreases in the sample size, whereas the linewidth decreased. The relaxation time for sample A was 37.2779 ps, which increased to 42.8301 ps for sample D; this is evident from the broad tail in the smaller size samples. The detailed characterization confirms the improved stability and magnetic characteristics of the AMFs. This makes them more sustainable for biomedical applications such as targeted drug delivery, magnetic resonance imaging (MRI), and hyperthermia treatment. 

## Figures and Tables

**Table 1:** Crystallite size, strain, various refinement parameters (goodness of fit, R-factor), lattice parameters, and unit cell volume of the AMF samples.

Figure 2	FWHM (Degree)	Crystallite Size (nm) D-S Method	W-H Method		χ^2^	Bragg R-Factor	RF-Factor (Crystallographic Factor)	R^wp^ (Discrepancy Factor)	R^exp^ (Expected Value)	R_P_	Lattice Parameter (a = b = c) (Å)	Unit Cell Volume (Å)^3^
			Crystallite Size (nm)	Strain								
A	0.83	9.73	10.6	0.0020	1.43	1.356	1.008	54.8	47.26	133	8.3482	581.81
B	0.86	9.32	10.1	0.0026	1.23	1.097	0.782	56.8	50.12	117	8.3460	581.37
C	1.03	7.46	8.5	0.0060	1.23	1.154	0.745	65.4	48.33	120	8.3451	581.16
D	1.51	5.32	5.8	0.0150	1.42	1.334	0.998	45.8	47.93	115	8.3440	580.93

**Table 2:** Comparison of the average size obtained from the SAXS and TEM for the AMF samples A, B, C, and D.

Sample	Average Diameter (SAXS) (nm)	Average Diameter (TEM) (nm)	Resize Distribution
A	16.6	17.2	51.08
B	12.3	10.2	65.08
C	9.30	8.3	57.9
D	7.2	7.5	63.7

**Table 3:** The dc magnetic properties (saturation magnetization, coercivity, and remanence) and Langevin fitting parameters (domain magnetization (*M_d_*) and permeability (*μ_m_*)), anisotropy constant, standard deviation (*σ_m_*), and hydrodynamic diameter of AMF samples.

Sample	*M_s_* (emu/g)	*H_c_* (G)	*M_r_* (emu/g)	*k_anis_* (erg/cm^3^)	*μ_m_* (10^−19^ Am^2^)	Hydrodynamic Diameter (nm)	*M_d_* (10^5^ A/m)	*σ_m_*
A	54.5	52.44	2.18	27,682.0	1.05	12.06	2.80	0.70
B	50.2	51.36	1.98	32,383.1	1.10	11.72	3.40	0.80
C	46.3	28.69	1.33	16,684.0	0.95	10.70	4.20	0.84
D	40.1	21.92	1.27	11,040.1	0.9	7.25	4.70	0.86

**Table 4:** Various spin resonance parameters calculated by fitting the FMR spectra of the AMF samples A, B, C, and D.

Sample	Crystallite Size (nm)	*g*-Value	Resonance Field, *H_R_* (G)	Δ*H_pp_* (G)	Δ*H*_1/2_ (G)	Relaxation Time, *T*_2_ (×10^−12^ s)	Spin Concentration *N_s_* (×10^22^ Spins/G)
A	10.6	2.236	3150.51	1188.66	1364.22	37.2779	1499.665
B	10.1	2.159	3262.246	1180.84	1377.68	38.2303	1568.469
C	8.5	2.156	3267.196	1164.95	1276.35	41.3226	1455.136
D	5.8	2.109	3338.789	1102.64	1254.60	42.8301	1462.217

## References

[1] Sheoran N., Kumar V., Kumar A. (2019). Comparative study of structural, magnetic and dielectric properties of CoFe_2_O_4_ @ BiFeO_3_ and BiFeO3@ CoFe_2_O_4_ core-shell nanocomposites. J. Magn. Magn. Mater..

[2] Kumar P., Sharma V., Singh J.P., Kumar A., Chahal S., Sachdev K., Chae K.H., Kumar A., Asokan K., Kanjilal D. (2019). Investigations on magnetic and electrical properties of Zn doped Fe_2_O_3_ nanoparticles and their correlation with local electronic structures. J. Magn. Magn. Mater..

[3] Mizuguchi M., Nakatsuji S. (2019). Energy-harvesting materials based on the anomalous Nernst effect. Sci. Technol. Adv. Mater..

[4] Kumar P., Pathak S., Singh A., Kuldeep Khanduri H., Wang X., Basheed G.A., Pant R.P. (2021). Optimization of cobalt concentration for improved magnetic characteristics and stability of CoxFe_3_-xO_4_ mixed ferrite nanomagnetic fluids. Mater. Chem. Phys..

[5] Victory M., Pant R.P., Phanjoubam S. (2020). Synthesis and characterization of oleic acid coated Fe–Mn ferrite based ferrofluid. Mater. Chem. Phys..

[6] Joshi L.M., Verma A., Rout P.K., Kaur M., Gupta A., Budhani R.C. (2017). The 2D–3D crossover and anisotropy of upper critical fields in Nb and NbN superconducting thin films. Phys. C Supercond. Appl..

[7] Phor L., Chahal S., Kumar V. (2020). Zn^2+^ substituted superparamagnetic MgFe_2_O_4_ spinel-ferrites: Investigations on structural and spin-interactions. J. Adv. Ceram..

[8] Gbadamasi S., Mohiuddin M., Krishnamurthi V., Verma R., Khan M.W., Pathak S., Kalantar-Zadeh K., Mahmood N. (2021). Interface chemistry of two-dimensional heterostructures—Fundamentals to applications. Chem. Soc. Rev..

[9] Sharma P., Alekhya V.V., Pathak S., Jain K., Tomar P., Basheed G.A., Maurya K.K., Pant R.P. (2021). A novel experimental approach for direct observation of magnetic field induced structuration in ferrofluid. J. Magn. Magn. Mater..

[10] Chahal S., Kumar A., Kumar P. (2020). Zn Doped α-Fe_2_O_3_: An Efficient Material for UV Driven Photocatalysis and Electrical Conductivity. Crystals.

[11] Basheed G.A., Jain K., Pathak S., Pant R.P. (2018). Dipolar Interaction and Magneto-Viscoelasticity in Nanomagnetic Fluid. J. Nanosci. Nanotechnol..

[12] Mousavi N.S.S., Khapli S.D., Kumar S. (2015). Direct observations of field-induced assemblies in magnetite ferrofluids. J. Appl. Phys..

[13] Pathak S., Jain K., Kumar V., Pant R.P. (2017). Magnetic Fluid Based High Precision Temperature Sensor. IEEE Sens. J..

[14] Phor L., Kumar V. (2019). Self-cooling by ferrofluid in magnetic field. SN Appl. Sci..

[15] Jain K., Pathak S., Pant R.P. (2016). Enhanced magnetic properties in ordered oriented ferrofibres. RSC Adv..

[16] Jain K., Pathak S., Kumar P., Singh A., Pant R.P. (2019). Dynamic magneto-optical inversion in magnetic fluid using NanoMOKE. J. Magn. Magn. Mater..

[17] Jahan N., Pathak S., Jain K., Pant R.P. (2017). Enchancment in viscoelastic properties of flake-shaped iron based magnetorheological fluid using ferrofluid. Colloids Surf. A Physicochem. Eng. Asp..

[18] Pathak S., Jain K., Pant R.P. (2018). Improved magneto-viscoelasticity of cross-linked PVA hydrogels using magnetic nanoparticles. Colloids Surf. A Physicochem. Eng. Asp..

[19] Pathak S., Verma R., Singhal S., Chaturvedi R., Kumar P., Sharma P., Pant R.P., Wang X. (2021). Spin dynamics investigations of multifunctional ambient scalable Fe_3_O_4_ surface decorated ZnO magnetic nanocomposite using FMR. Sci. Rep..

[20] Pathak S., Jain K., Kumar P., Wang X., Pant R.P. (2019). Improved thermal performance of annular fin-shell tube storage system using magnetic fluid. Appl. Energy.

[21] Mishra A., Pathak S., Kumar P., Singh A., Jain K., Chaturvedi R., Singh D., Basheed G.A., Pant R.P. (2019). Measurement of Static and Dynamic Magneto-Viscoelasticity in Facile Varying pH Synthesized CoFe_2_O_4_-Based Magnetic Fluid. IEEE Trans. Magn..

[22] Mirkhani N., Christiansen M.G., Schuerle S. (2020). Living, Self-Replicating Ferrofluids for Fluidic Transport. Adv. Funct. Mater..

[23] Genc S., Derin B. (2014). Synthesis and rheology of ferrofluids: A review. Curr. Opin. Chem. Eng..

[24] Radha S., Mohan S., Pai C. (2014). Diffraction patterns in ferrofluids: Effect of magnetic field and gravity. Phys. B Condens. Matter.

[25] Clark N.A. (2013). Ferromagnetic ferrofluids. Nature.

[26] Fang A. (2020). Generic theory of the dynamic magnetic response of ferrofluids. Soft Matter.

[27] Petrenko V.I., Artykulnyi O.P., Bulavin L.A., Almásy L., Garamus V.M., Ivankov O.I., Grigoryeva N.A., Vekas L., Kopcansky P., Avdeev M.V. (2018). On the impact of surfactant type on the structure of aqueous ferrofluids. Colloids Surf. A Physicochem. Eng. Asp..

[28] Verma R., Pathak S., Srivastava A.K., Prawer S., Tomljenovic-Hanic S. (2021). ZnO nanomaterials: Green synthesis, toxicity evaluation and new insights in biomedical applications. J. Alloys Compd..

[29] Sharifianjazi F., Moradi M., Parvin N., Nemati A., Jafari Rad A., Sheysi N., Abouchenari A., Mohammadi A., Karbasi S., Ahmadi Z. (2020). Magnetic CoFe_2_O_4_ nanoparticles doped with metal ions: A review. Ceram. Int..

[30] Pathak S., Zhang R., Bun K., Zhang H., Gayen B., Wang X. (2021). Development of a novel wind to electrical energy converter of passive ferrofluid levitation through its parameter modelling and optimization. Sustain. Energy Technol. Assess..

[31] Mallick A., Mahapatra A.S., Mitra A., Greneche J.M., Ningthoujam R.S., Chakrabarti P.K. (2018). Magnetic properties and bio-medical applications in hyperthermia of lithium zinc ferrite nanoparticles integrated with reduced graphene oxide. J. Appl. Phys..

[32] Shokrollahi H. (2017). A review of the magnetic properties, synthesis methods and applications of maghemite. J. Magn. Magn. Mater..

[33] Verma R., Gangwar J., Srivastava A.K. (2017). Multiphase TiO_2_ nanostructures: A review of efficient synthesis, growth mechanism, probing capabilities, and applications in bio-safety and health. RSC Adv..

[34] Li J., Khalid A., Verma R., Abraham A., Qazi F., Dong X., Liang G., Tomljenovic-Hanic S. (2021). Silk Fibroin Coated Magnesium Oxide Nanospheres: A Biocompatible and Biodegradable Tool for Noninvasive Bioimaging Applications. Nanomaterials.

[35] Wang Y., Miao Y., Li G., Su M., Chen X., Zhang H., Zhang Y., Jiao W., He Y., Yi J. (2020). Engineering ferrite nanoparticles with enhanced magnetic response for advanced biomedical applications. Mater. Today Adv..

[36] Hergt R., Dutz S. (2007). Magnetic particle hyperthermia—Biophysical limitations of a visionary tumour therapy. J. Magn. Magn. Mater..

[37] László J., Reiczigel J., Székely L., Gasparics A., Bogár I., Bors L., Rácz B., Gyires K. (2007). Optimization of static magnetic field parameters improves analgesic effect in mice. Bioelectromagnetics.

[38] Koo O.M., Rubinstein I., Onyuksel H. (2005). Role of nanotechnology in targeted drug delivery and imaging: A concise review. Nanomed. Nanotechnol. Biol. Med..

[39] Pathak S. (2020). Optimization of Magneto-Viscoelasticity of Magnetic Fluids and Development of Its Applications in Thermal and Mechanical Systems.

[40] Cao Q., Zhang Z., Yu J., Di N., Zang G., Li D. (2020). Research on the effect of different surfactants on fluidity of water-based magnetic fluid. Smart Mater. Struct..

[41] Dai Z., Huang Y., Yang H., Yao P., Yang Y., Ni C. (2018). Preparation and Biological Applications of Graphene Oxide Functionalized Water-Based Magnetic Fluids. J. Nanosci. Nanotechnol..

[42] Morales M.A., Jain T.K., Labhasetwar V., Leslie-Pelecky D.L. (2005). Magnetic studies of iron oxide nanoparticles coated with oleic acid and Pluronic® block copolymer. J. Appl. Phys..

[43] Bica D., Vékás L., Avdeev M.V., Marinică O., Socoliuc V., Bălăsoiu M., Garamus V.M. (2007). Sterically stabilized water based magnetic fluids: Synthesis, structure and properties. J. Magn. Magn. Mater..

[44] Vékás L., Bica D., Avdeev M.V. (2007). Magnetic nanoparticles and concentrated magnetic nanofluids: Synthesis, properties and some applications. China Particuol..

[45] Kuncser V., Schinteie G., Sahoo B., Keune W., Bica D., Vekas L., Filoti G. (2006). Magnetic interactions in water based ferrofluids studied by Mössbauer spectroscopy. J. Phys. Condens. Matter.

[46] Kumar P., Khanduri H., Pathak S., Singh A., Basheed G.A., Pant R.P. (2020). Temperature selectivity for single phase hydrothermal synthesis of PEG-400 coated magnetite nanoparticles. Dalton Trans..

[47] Kraus L., Vázquez M. (2015). 15-Ferromagnetic resonance in individual wires: From micro- to nanowires. Magnetic Nano- and Microwires.

[48] Kumar P., Pathak S., Singh A., Khanduri H., Jain K., Tawale J., Wang L., Basheed G.A., Pant R.P. (2021). Enhanced static and dynamic magnetic properties of PEG-400 coated CoFe_2_-xErxO_4_ (0.7 ≤ x ≤ 0) nanoferrites. J. Alloys Compd..

[49] Singh A., Pathak S., Kumar P., Sharma P., Rathi A., Basheed G.A., Maurya K.K., Pant R.P. (2020). Tuning the magnetocrystalline anisotropy and spin dynamics in CoxZn1-xFe_2_O_4_ (0 ≤ x ≤ 1) nanoferrites. J. Magn. Magn. Mater..

[50] Zhang J., Yan S., Yuan D., Zhao Q., Tan S.H., Nguyen N.-T., Li W. (2016). A novel viscoelastic-based ferrofluid for continuous sheathless microfluidic separation of nonmagnetic microparticles. Lab. A Chip.

[51] Chirikov D.N., Fedotov S.P., Iskakova L.Y., Zubarev A.Y. (2010). Viscoelastic properties of ferrofluids. Phys. Rev. E.

[52] Saldivar-Guerrero R., Richter R., Rehberg I., Aksel N., Heymann L., Rodriguez-Fernández O.S. (2006). Viscoelasticity of mono- and polydisperse inverse ferrofluids. J. Chem. Phys..

[53] Odenbach S. (2000). Magnetoviscous and Viscoelastic Effects in Ferrofluids. Int. J. Mod. Phys. B.

[54] De Gans B.J., Blom C., Philipse A.P., Mellema J. (1999). Linear viscoelasticity of an inverse ferrofluid. Phys. Rev. E.

[55] Odenbach S., Rylewicz T., Heyen M. (1999). A rheometer dedicated for the investigation of viscoelastic effects in commercial magnetic fluids. J. Magn. Magn. Mater..

[56] Odenbach S., Thurm S., Odenbach S. (2002). Magnetoviscous Effects in Ferrofluids. Ferrofluids: Magnetically Controllable Fluids and Their Applications.

[57] Kumar P., Pathak S., Singh A., Khanduri H., Basheed G.A., Wang L., Pant R.P. (2020). Microwave spin resonance investigation on the effect of post processing annealing of CoFe_2_O_4_ nanoparticles. Nanoscale Adv..

[58] Rosensweig R.E. (2016). Ferrofluids: Introduction. Reference Module in Materials Science and Materials Engineering.

[59] Anupama A.V., Keune W., Sahoo B. (2017). Thermally induced phase transformation in multi-phase iron oxide nanoparticles on vacuum annealing. J. Magn. Magn. Mater..

[60] Jagadeesha Angadi V., Anupama A.V., Kumar R., Matteppanavar S., Rudraswamy B., Sahoo B. (2016). Observation of enhanced magnetic pinning in Sm^3+^ substituted nanocrystalline MnZn ferrites prepared by propellant chemistry route. J. Alloys Compd..

[61] Goswami L., Aggarwal N., Verma R., Bishnoi S., Husale S., Pandey R., Gupta G. (2020). Graphene Quantum Dot-Sensitized ZnO-Nanorod/GaN-Nanotower Heterostructure-Based High-Performance UV Photodetectors. ACS Appl. Mater. Interfaces.

[62] Verma R., Singh S., Dalai M.K., Saravanan M., Agrawal V.V., Srivastava A.K. (2017). Photocatalytic degradation of polypropylene film using TiO_2_-based nanomaterials under solar irradiation. Mater. Des..

[63] Marwaha N., Gupta B.K., Verma R., Srivastava A.K. (2017). Facile synthesis and characterization of pH-dependent pristine MgO nanostructures for visible light emission. J. Mater. Sci..

[64] Verma R., Awasthi A., Singh P., Srivastava R., Sheng H., Wen J., Miller D.J., Srivastava A.K. (2016). Interactions of titania based nanoparticles with silica and green-tea: Photo-degradation and -luminescence. J. Colloid Interface Sci..

[65] Verma R., Naik K.K., Gangwar J., Srivastava A.K. (2014). Morphology, mechanism and optical properties of nanometer-sized MgO synthesized via facile wet chemical method. Mater. Chem. Phys..

[66] Chen P., Zhang Z., Duan X., Duan X. (2018). Chemical synthesis of two-dimensional atomic crystals, heterostructures and superlattices. Chem. Soc. Rev..

[67] Goswami L., Aggarwal N., Singh M., Verma R., Vashishtha P., Jain S.K., Tawale J., Pandey R., Gupta G. (2020). GaN Nanotowers Grown on Si(111) and Functionalized with Au Nanoparticles and ZnO Nanorods for Highly Responsive UV Photodetectors. ACS Appl. Nano Mater..

[68] Verma R., Chaudhary V.B., Nain L., Srivastava A.K. (2017). Antibacterial characteristics of TiO_2_ nano-objects and their interaction with biofilm. Mater. Technol..

[69] Okuda M., Eloi J.-C., Ward Jones S.E., Sarua A., Richardson R.M., Schwarzacher W. (2012). Fe_3_O_4_ nanoparticles: Protein-mediated crystalline magnetic superstructures. Nanotechnology.

[70] Suzuki N., Gupta P., Sukegawa H., Inomata K., Inoue S., Yamauchi Y. (2010). Aerosol-Assisted Synthesis of Thiol-Functionalized Mesoporous Silica Spheres with Fe_3_O_4_ Nanoparticles. J. Nanosci. Nanotechnol..

[71] Doniach S., Lipfert J. (2009). Chapter 11—Use of Small Angle X-ray Scattering (SAXS) to Characterize Conformational States of Functional RNAs. Methods in Enzymology.

[72] Balasoiu M., Erhan R., Craus M.L., Plestil J., Haramus V., Lozovan M., Kuklin A.I., Bica I. (2008). Microstructure of Magnetite Doped Elastomers Investigated by SAXS and SANS.

[73] Jain N., Marwaha N., Verma R., Gupta B.K., Srivastava A.K. (2016). Facile synthesis of defect-induced highly-luminescent pristine MgO nanostructures for promising solid-state lighting applications. RSC Adv..

[74] Zhang X., Sun L., Yu Y., Zhao Y. (2019). Flexible Ferrofluids: Design and Applications. Adv. Mater..

[75] Kalaiselvan C.R., Thorat N.D., Sahu N.K. (2021). Carboxylated PEG-Functionalized MnFe_2_O_4_ Nanocubes Synthesized in a Mixed Solvent: Morphology, Magnetic Properties, and Biomedical Applications. ACS Omega.

[76] Kopyl S., Timopheev A.A., Bystrov V.S., Bdikin I., Teixeira B.M.S., Maevskij E., Sobolev N.A., Sousa A.C.M. (2014). FMR study of carbon nanotubes filled with Fe_3_O_4_ nanoparticles. J. Magn. Magn. Mater..

[77] Kurlyandskaya G.V., Cunanan J., Bhagat S.M., Aphesteguy J.C., Jacobo S.E. (2007). Field-induced microwave absorption in Fe_3_O_4_ nanoparticles and Fe_3_O_4_/polyaniline composites synthesized by different methods. J. Phys. Chem. Solids.

[78] Shankar A., Chand M., Basheed G.A., Thakur S., Pant R.P. (2015). Low temperature FMR investigations on double surfactant water based ferrofluid. J. Magn. Magn. Mater..

[79] Owens F.J. (2003). Ferromagnetic resonance of magnetic field oriented Fe_3_O_4_ nanoparticles in frozen ferrofluids. J. Phys. Chem. Solids.

[80] Dixit G., Pal Singh J., Srivastava R.C., Agrawal H.M. (2012). Magnetic resonance study of Ce and Gd doped NiFe_2_O_4_ nanoparticles. J. Magn. Magn. Mater..

[81] Wu K.H., Yu C.H., Chang Y.C., Horng D.N. (2004). Effect of pH on the formation and combustion process of sol–gel auto-combustion derived NiZn ferrite/SiO_2_ composites. J. Solid State Chem..

[82] Noginova N., Weaver T., Andreyev A., Radocea A., Atsarkin V.A. (2009). NMR and spin relaxation in systems with magnetic nanoparticles: Effects of size and molecular motion. J. Phys. Condens. Matter.

[83] Yin J., Xu F., Qu H., Li C., Liu S., Liu L., Shao Y. (2019). Dysprosium-doped iron oxide nanoparticles boosting spin–spin relaxation: A computational and experimental study. Phys. Chem. Chem. Phys..

